# Role of Mangiferin in Management of Cancers through Modulation of Signal Transduction Pathways

**DOI:** 10.3390/biomedicines11123205

**Published:** 2023-12-01

**Authors:** Arshad Husain Rahmani, Ahmad Almatroudi, Khaled S. Allemailem, Hajed Obaid A. Alharbi, Wanian M. Alwanian, Basmah Awwadh Alhunayhani, Mohammad Algahtani, Abdulrahman Theyab, Nahlah Makki Almansour, Ahmed N. Algefary, Solaiman Saleh Ali Aldeghaim, Amjad Ali Khan

**Affiliations:** 1 Department of Medical Laboratories, College of Applied Medical Sciences, Qassim University, Buraydah 51452, Saudi Arabiahajed.alharbi@qu.edu.sa (H.O.A.A.); ah.algefary@qu.edu.sa (A.N.A.); so.aldeghaim@qu.edu.sa (S.S.A.A.); 2 Department of Laboratory & Blood Bank, Security Forces Hospital, P.O. Box 14799, Mecca 21955, Saudi Arabia; 3 College of Medicine, Al-Faisal University, P.O. Box 50927, Riyadh 11533, Saudi Arabia; 4 Department of Biology, College of Science, University of Hafr Al Batin, Hafr Al Batin 31991, Saudi Arabia; 5 Department of Basic Health Sciences, College of Applied Medical Sciences, Qassim University, Buraydah 51452, Saudi Arabia

**Keywords:** mangiferin, inflammation, pathogenesis, cancer therapy, signal transduction pathways

## Abstract

Cancer is a major public health concern worldwide in terms of mortality. The exact reason behind the development of cancer is not understood clearly, but it is evidenced that alcohol consumption, radiation, and exposure to chemicals are main players in this pathogenesis. The current mode of treatments such as surgery, chemotherapy, and radiotherapy are effective, but, still, cancer is a major problem leading to death and other side effects. However, safer and effective treatment modules are needed to overcome the adverse effects of current treatment modules. In this regard, natural compounds have been recognized to ameliorate diseases by exerting anti-inflammatory, anti-oxidative, and anti-tumor potential through several mechanisms. Mangiferin, a xanthone C-glucoside, is found in several plant species including *Mangifera indica* (mango), and its role in disease prevention has been confirmed through its antioxidant and anti-inflammatory properties. Furthermore, its anti-cancer-potential mechanism has been designated through modulation of cell signaling pathways such as inflammation, angiogenesis, PI3K/AKT, apoptosis, and cell cycle. This article extensively reviews the anticancer potential of mangiferin in different cancers through the modulation of cell signaling pathways. Moreover, the synergistic effects of this compound with some commonly used anti-cancer drugs against different cancer cells are discussed. More clinical trials should be performed to reconnoiter the anti-cancer potential of this compound in human cancer treatment. Further, understanding of mechanisms of action and the safety level of this compound can help to manage diseases, including cancer.

## 1. Introduction

Cancer is a main culprit of death and a burden on the healthcare system worldwide. As per the projections of the World Health Organization (WHO), cancer is the first or second principal cause of death before the age of 70 years in 112 of 183 countries, as well as placing third or fourth in another twenty-three countries [1]. Further, in accordance with the International Agency for Research on Cancer, a global growth of 10.9 million cancer cases as well, as 6.3 million cancer deaths, is predicted by 2040 due to demographic changes [2]. During the last decade, this incidence increased by 33%, with population aging contributing 16%, population growth adding 13%, and changes in age-specific rates contributing 4% [3]. The exact reason behind the growth of cancer is not understood clearly, but it is supposed that alcohol consumption, radiation, chemicals, and smoking are main players in this pathogenesis.

These days, various types of treatment modules are used to treat cancer, including surgery, chemotherapy, and radiotherapy, but still, cancer stands to be a major problem worldwide. Moreover, treatment strategies like chemotherapy and radiotherapy are known to exhibit adverse side effects [4,5]. However, safe and effective treatment modules are needed to overcome the adverse effects of commonly used anticancer drugs. Previous studies evidence that natural compounds play a role in diseases management, including for cancer [6,7,8,9].

**Figure 1:**
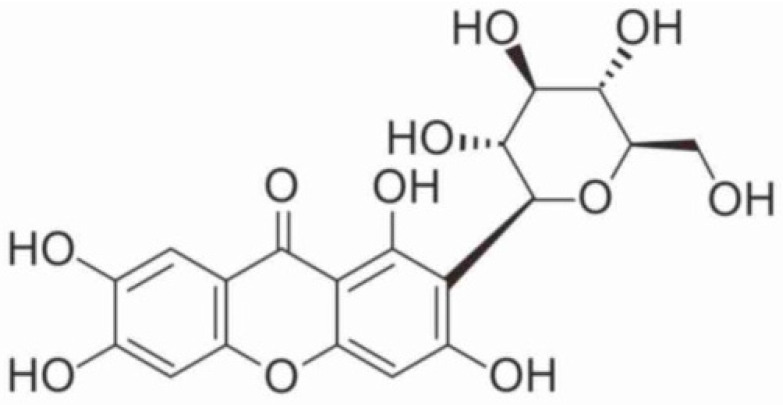
Chemical structure of mangiferin.

Mangiferin, structurally termed 2-C-β-D-glucopyranosyl-1, 3, 6, 7-tetrahydroxyxanthone, is a powerful medicinal compound isolated from widespread natural sources from plants and fruits (Figure 1) [10].

Its health-promoting potential has been noticed through modulation of various biological activities. Moreover, it has been broadly described that mangiferin offers an extensive plethora of useful potential such as hepatoprotective, antioxidant, and cytotoxic effects [11,12]. Furthermore, the anti-cancer activity of mangiferin is documented through modulation of cell signaling pathways [13]. Furthermore, its synergistic role with anticancer drugs has been reported through the enhancement of anti-cancer drug efficacies [14]. This article reviews the anti-cancerous potential of mangiferin in numerous malignancies and measures the possible anti-cancer effects through modulating cell-signaling pathways. In addition, its synergistic effects with anti-cancer drugs and strategies to improve its efficacies are discussed accordingly.

## 2. Methodology

Two members independently searched the electronic databases Google Scholar, PubMed, Scopus, Embase, Cochrane, Medline, and Web of Science to collect information based on mangiferin. The keywords used to search scientific content were mangiferin sources, mechanism of action, cell-signaling pathways, and role in different cancer, bioavailability, and synergistic effects.

## 3. Mechanism of Action of Mangiferin in Cancer Management

**Figure 2:**
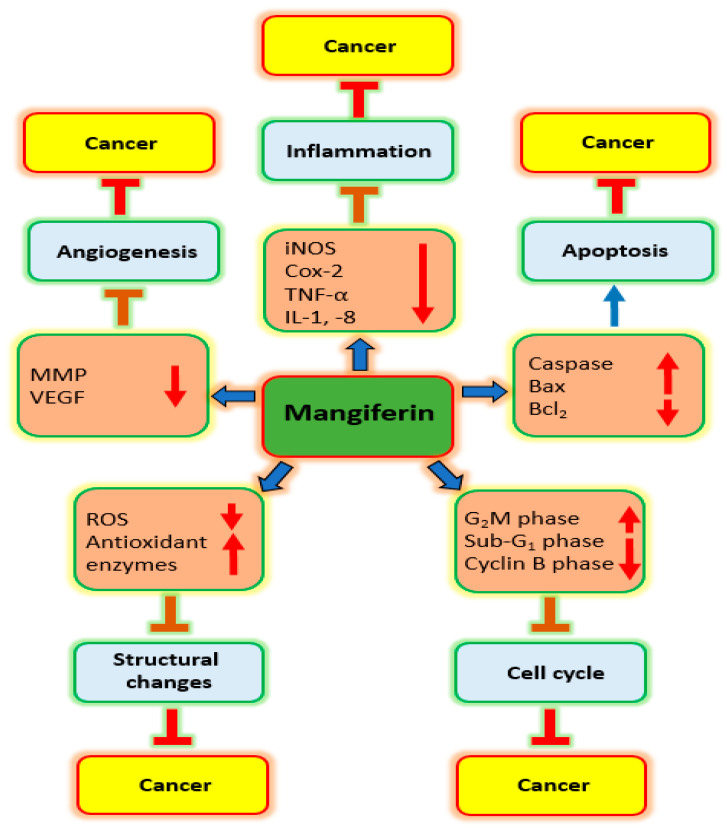
Role of mangiferin in different malignancies through modulation of cell signaling pathways.

Mangiferin plays a role in managing cancer via intonation of cell signaling pathways (Figure 2).

The principal mechanisms of action by which mangiferin shows anti-cancer potential are inhibition of inflammation, oxidative stress, apoptosis and angiogenesis, and cell cycle and PI3K/Akt pathways as discussed below:

### 3.1. Inflammation

Inflammation has been established to be closely linked with all stages of the development and progression of cancer [15,16,17]. Around 25% of cancer cases have been estimated to be associated with chronic inflammation [18]. Moreover, cancer cells release more cytokines as well as chemokines, which call in immune cells and aggravate inflammation further [19].

The interest in the direction of use of natural compounds as well as their derivatives has been transformed, resulting in new findings as well as the development of new drugs [20]. Several natural compounds and their derivatives have proven their role in the inhibition of pathogenesis through their anti-inflammatory potential [21,22,23,24,25] (Table 1).

In one study, it has been reported that culture of *H. pylori*-infected gastric-cancer AGS cells with mangiferin increased the inhibitory zone and considerably lowered the levels of MBC and MIC. In the meantime, inflammatory markers such as interleukin-1β, IL-8, and NF-κΒ subunit p65 were also evidently decreased by the mangiferin. Furthermore, the protein expression of inflammatory enzymes such as iNOS and COX-2 were remarkably downregulated in the same cells incubated with mangiferin. Additionally, *H. pylori*-infected gastric cancer cell lines (AGS) treated with mangiferin significantly inhibited the adhesion and invasion process and deactivated NF-p65 (*p* < 0.05), hence inhibiting an inflammatory response and lowering the incidence of gastric carcinoma [26].

Translocation of the nuclear factor kappa B (NF-kB) to the nucleus and the expression of phosphorylated inhibitor kappa B (IkB) were both inhibited by mangiferin, whereas IkB protein expression was elevated. Additionally, mangiferin inhibits NIK activation in multiple myeloma cell lines, which causes apoptosis by preventing NF-kB translocation [36]. A pioneer study based on melanoma reported that mangiferin suppressed the nuclear translocation of NF-κB, as well as the expression of pNF-κB-inducing kinase. Furthermore, it is reported that mangiferin suppresses the matrix metalloproteinases (MMPs) expression and very-late antigens in vivo. The NF- κB pathway is suppressed using the mangiferin treatment through the inhibition of NIK activation, thus preventing metastasis and tumor growth [37].

Treatment with different dosages of mangiferin, the TNF-α, IL-1β, IL-8, and IL-6 were meaningfully reduced (*p* < 0.01) [38]. Moreover, mangiferin improves LPS-induced histopathology, and, in the meantime, also reduced LPS-induced MPO activity. Furthermore, mangiferin initiated the inhibition of LPS-initiated NF-ĸB, as well as NLRP3 inflammasome activation [39]. Mangiferin (100 mg/kg) decreased TNF-α as well as IL-17 levels in colon tissues [40].

The NF-κB assay on oxaliplatin-resistant HT29 cells demonstrated an enhanced amount of activated NF-κB than normal HT29 cells. However, this amount was reduced to less than normal control cells. Thus, mangiferin decreases the level of activated NF-κB associated with resistant tumor cells [41]. 

Another study reported that at the molecular level, mangiferin and gallic acid both inhibit NF-κB activation by IKKα/β kinases, which results in impaired NF-κB translocation, IκB degradation, and NF-κB/DNA binding. In contrast to the xanthone mangiferin, gallic acid additionally prevents NF-κB pathways participating in cancer cell survival and therapy resistance [42].

The result based on prostate cancer showed that mangiferin inverted TNF-α-induced mRNA as well as MMP-9 expression. Moreover, it is reported that mangiferin reduced TNF-α-induced invasion of cancer cells. Further, mangiferin treatment reduced TNF-α-induced NF-κB luciferase activity and reduced translocation of NF-κB subunits p65 and p50 to the nucleus [32].

### 3.2. Oxidative Stress

Oxidative stress plays a significant role in the development of several metabolic and chronic diseases or cancers [43,44]. Cancer cells display abnormal redox homeostasis, where the reactive oxygen species (ROS) are pro-tumorigenic and higher ROS levels lead to cytotoxicity [45].

Xanthine oxidase and the advance glycation end product (AGE) play a vital role in inducing ROS; however, treatment with mangiferin minimized the AGE generation and activities of xanthine oxidase [46]. Thus, mangiferin decreases the ROS level and reduces the damage of antioxidants, and, therefore, recovering sepsis-linked organ damage [47].

It was investigated whether mangiferin has any protective effects against the genotoxicity caused by cadmium chloride (CdCl_2_). Mangiferin pretreatment dramatically increased the activity of the glutathione-S-transferase, catalase, superoxide dismutase, and glutathione while decreasing lipid peroxidation in the liver. A study revealed that mangiferin has a potent antigenotoxic effect against cadmium chloride-caused toxicity, which might be due to increased antioxidant status and scavenging of free radicals [48]. Mangiferin has neuro-cyto-protective effects associated, at least in part, with an antioxidant as well as anti-inflammatory mechanism, which could be revealed as having an impact for more powerful remedies for schizophrenia as well as other neurodegenerative pathogenesis [49].

The role of mangiferin treatment on intracellular oxidative stress was investigated. The findings revealed that etoposide stimulation increased intracellular reactive oxygen species in human myeloid leukemia HL-60 cells as compared with the control. Though, when these cancer cells were exposed with mangiferin prior to etoposide incubation, intracellular reactive oxygen species levels were lower than in the cells incubated with etoposide only. Intracellular reactive oxygen species levels were meaningfully lower in cancer cells treated with mangiferin only rather than in the cells without any treatment [35].

The role of mangiferin in the prevention of lung carcinogenesis is reportedly through the reduction of oxidative stress, as levels of antioxidant enzymes were decreased in the Benzopyrene (BaP)-treated group. However, in the mangiferin + BaP-treated animals, GSH and antioxidant enzymes were increased in comparison with BaP-induced animals [50].

Another study based on lung carcinogenesis reported that decreased antioxidant enzymes and enhanced lipid peroxidation activities noticed in the polymorphonuclear cells, lymphocytes, and macrophages of B(a)P-treated animals were increased and reduced, correspondingly, by the treatment of mangiferin [51].

Moreover, detoxifying enzymes, for example uridin-5′-diphosphate-glucuronosyl transferase, glutathione transferase, and quinone reductase, were noticed to be decreased, whereas the lipid peroxidation level was enhanced in the lung-cancer-bearing animals. Administration of mangiferin increased the detoxification enzymes and decreased DNA damage. This finding elucidates the unique association between the antioxidant effect of mangiferin and, finally, the ability of mangiferin to prevent cancer [52].

### 3.3. Cell Cycle

Cell-cycle development is chiefly facilitated by cyclin-dependent kinases (CDKs) [53]. The cyclin subunit of CDK leads to site-specific phosphorylation, activation, and allows the cell to enter a cell-cycle phase [54]. Cell-cycle checkpoints are scrutiny mechanisms in eukaryotic cells that permit the repair of cellular damage in response to stress [55]. Altered cell-cycle progression has been noticed in cancer. In this regard, natural compounds and their derivatives play a significant role in cancer through the regulation of cell cycles. Mangiferin had a significant effect on the suppression of leukemia-cell proliferation. Cells in the G2/M phase increased after 24 h of treatment, and G2/M-phase arrest was observed. Mangiferin also increased the expression of Wee1 mRNA in dosage-dependent ways, whereas at high concentrations it reduced the expression of Chk1 and cdc25c mRNA. It meaningfully increased cdc2 and cyclin B1 phosphorylation and prevented Wee1, Akt, ATR, Chk1, and ERK1/2 phosphorylation. Furthermore, mangiferin induces Wee1 protein expression and decreases cdc25c, cyclin B1, and Akt protein levels [27].

In order to determine how lung cancer cells responded to mangiferin treatment, cell cycle analysis was performed. Analysis of the same cells at sub-G_1_, G_0_/G_1_, S as well as G_2_/M phase demonstrated that they were arrested in the sub-G1 phase after mangiferin administration, indicating that this compound may stimulate the apoptosis of cancer cells [28] (Table 1).

The growth-inhibition effects of mangiferin in leukemia cells were increased as the mangiferin concentration enhanced and exposure time continued; leukemia HL-60 cells in the G2/M phase increased in a dose-dependent way after administration of mangiferin, demonstrating G2/M-phase blockage [56]. Mangiferin stimulated autophagy in pancreatic cancer cells, as evidenced by increased expression of LC3 II and Beclin-1. Mangiferin antiproliferative potential was also challenged through the production of endogenous ROS and cell-cycle arrest [57].

### 3.4. Apoptosis

Different cancer hallmarks such as angiogenesis, uncontrolled growth, and apoptosis evasion are identified in all cancer cells [58,59], and apoptosis mainly proceeds through two main pathways [60]. Different anticancer agents have been reported in natural products explored as effective chemotherapeutic cancer drugs [61].

Natural products have a proven effect in cancer treatment by modulating apoptosis. Caspases-3 and apoptotic activity have been investigated to assess the role of mangiferin (0 to 40 µM) on PC3 cell apoptosis. Mangiferin promoted cancer cell death and caspase-3 activity in a concentration-dependent manner. Additionally, mangiferin (20 as well as 40 µM) treatment boosted the apoptosis of PC3 cells as compared to the control group that received mangiferin at a concentration of 0 µM. Furthermore, when compared to the control group, mangiferin treatment (20 and 40 µM) elevated the level of caspase-3 activity in PC3 cells [29] (Table 1).

Mangiferin-treated cells in nasopharyngeal cancer-based report showed the enhancement of Bax mRNA level and decrease of Bcl-2 mRNA level as compared to the control. Consistently, the protein level of Bax was upregulated and the protein level of Bcl-2 was downregulated in mangiferin-treated cells compared to the control [62]. Mangiferin (100 mg/kg body weight), given orally once per day, meaningfully increased antioxidant levels. Moreover, mangiferin inhibited NF-κBp65 nucleus transcriptional activation, suppressing inflammation as well as cell proliferation, and it increased pro-apoptotic proteins [63]. 

The cells exposed to 25 µg/mL mangiferin displayed shrinking of the cytoplasm and nucleus, and shattering and blebbing of the membrane, all of which are signs of apoptotic morphology. In the mangiferin-treated group, there were more early and late apoptotic cells than in the control group. Thus, mangiferin was capable of encouraging apoptosis in ovarian carcinoma cells. The cytosol and mitochondrial cytochrome c levels were measured, and it was discovered that mangiferin administration might promote cytochrome c translocation from mitochondria. Mangiferin treatment led to an increase in cleaved caspase-3 and -9 and its triggering [30] (Table 1). 

### 3.5. Angiogenesis

Angiogenesis is a multi-step process, activated using a variety of biological signals irrespective of physiological or pathological potential [64]. Angiogenesis delivers cancer cells with a vital supply of oxygen and nutrients, assisting their rapid growth and has a noteworthy impact in the progression and spread of cancers [65,66]. Mangiferin remarkably prevented HG/hypoxia-induced rat retinal capillary endothelial cell migration and angiogenesis [67]. Mangiferin selectively inhibits multiple NF-kB target genes by affecting inflammation, and lipid as well as calcium signaling. This blocks the formation of capillary tubes in human placental-blood-vessel explants, metastatic melanoma cells, and invasive processes in vitro. It also reduces the angiogenesis seen in vivo in melanoma syngeneic studies and the chicken egg chorioallantoic membrane assay [68] (Table 1). 

### 3.6. PI3K/Akt Pathway

The PI3K/AKT/mammalian target of rapamycin (mTOR) pathway hyperactivity is correlated with tumor progression in a wide variety of cancers [69]. Deregulation of PIK3CA mutations, AKT mutations or tensin homolog (PTEN) loss are the main reasons [70]. An enhanced mangiferin concentration resulted in decreased expression of p-PI3K, p-Akt, and p-mTOR. Additionally, SGC-7901 cells were treated with mangiferin and/or EGF to see if it had a direct opposite effect on the elevation of p-PI3K and p-Akt that was brought on by the EGF. EGF stimulation dramatically increased PI3K and Akt phosphorylation, while mangiferin administration inhibited EGF-activated phosphorylation of downstream p-PI3K and p-Akt (*p* < 0.01). Additionally, SC79 significantly raised p-Akt levels in SGC-7901 cells when together with mangiferin they had an antagonistic effect (*p* < 0.01) [31] (Table 1), and mangiferin inhibits the binding of NF-κB as well as AP-1 to the MMP-9 promoter and inhibits reductions of the PMA-initiated phosphorylation of Akt as well [71]. Additionally, mangiferin enhanced the expression of Wee1 mRNA in a dose-dependent manner, while inducing the expression of Chk1 and cdc25c at high dosages. It pointedly reduced the phosphorylation of Chk, ATR, Wee1, Akt, and ERK1/2 [27].

### 3.7. Invasion and Metastasis

**Figure 3:**
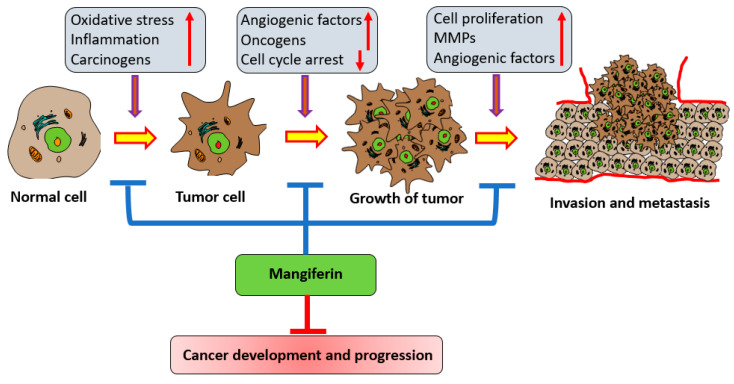
Role of mangiferin in inhibition of invasion and growth of cancer.

Tumor invasion and metastasis remain the most indescribable hallmark of cancer [72]. Furthermore, cell-extracellular matrix (ECM) interactions, degradation of the ECM, disconnection of intercellular adhesion, and the invasion of lymph and blood vessels are substantial steps in cancer invasion except for metastasis [73]. Epithelial-mesenchymal transition enables malignant cells to become motile and invasive, which establishes a fundamental essential for cancer metastasis [74]. Invasion and metastasis are central hallmarks of several cancers, and the majority of cancer-associated deaths are caused by metastatic disease rather than the primary tumor [75]. The possible role of mangiferin in cancer management has been noted through the inhibition of invasion and metastasis (Figure 3).

The stimulus of experimental cells with TNF-α enhanced the Matrix metallopeptidase 9 activity in prostate cancer cells. Although, mangiferin considerably decreased the TNF-α activity of Matrix metallopeptidase 9. Thus, mangiferin is likely to act as an anti-invasive agent that suppress NF-κB-mediated expression of MMP-9 [32]. Moreover, mangiferin promoted the expression of miR-15b, as well as reduced the level of glioma cell MMP-9 expression. MMP-9 agonist as well as anti-miR-15b decreases the restorative effects of mangiferin in the glioma cells [76].

Based on a breast cancer study, it was reported that mangiferin decreased the levels of matrix metalloproteinases-7 and -9 as well as the reversal of the epithelial-mesenchymal transition (EMT). The study further showed that modulating MMP-7 and -9 and EMT could lead to a central function for β-catenin pathway inhibition in mangiferin-induced anti-cancer activity [33] (Table 1).

### 3.8. NRF2 Transcription Factor

Nuclear factor erythroid 2-related factor 2 (NRF2), is a well-known transcription factor that guards cells from oxidative stress [77]. The double role of the NRF2-like tumor suppressor or driver depends on the tumor stage or type, and has given rise to a considerable debate about its role in malignancy [78]. In case of ROS accumulation, Nrf2 translocates into the nuclei, where it interacts with some protein factors, together with small Maf, and binds to the Antioxidant Responsive Element (ARE), leading to enhanced transcription of antioxidant genes and return redox homeostasis [78]. The HL60 cells were subjected to dose- and time-course studies with mangiferin to examine the effect of the compound on Nrf2 expression. Mangiferin was applied in doses of 50, 100, or 200 µM to HL60 cells. The Nrf2 protein level was raised by these therapies in a dose-dependent manner. Following treatment with 50 µM mangiferin, the Nrf2 level enhanced to almost 4-fold of the baseline value, and it increased to roughly 7.56-fold of the baseline value after being treated for 24 h with 200 µM mangiferin [34] (Table 1).

The increase of Nrf2 protein was examined to determine whether mangiferin promotes Nrf2-ARE signaling in HL-60 cells. The Nrf2 protein was present in the cytoplasm and nucleus of untreated cells. However, the cytoplasmic green fluorescence (which indicates Nrf2) was stronger in most cells, suggesting that the Nrf2 protein content was higher there than in the nucleus. After mangiferin administration, the levels of the Nrf2 protein in both the nuclear and overall tissues increased. According to the Western blot data, mangiferin boosted whole-cell accumulation of the Nrf2 protein in a manner that was dependent on both time as well as dose. Additionally, mangiferin administration increased the amount of Nrf2 protein in the cytoplasm and the nucleus compared to cells that were not treated with the drug [35] (Table 1).

## 4. Mangiferin: Potential Role in Several Types of Cancer

**Figure 4:**
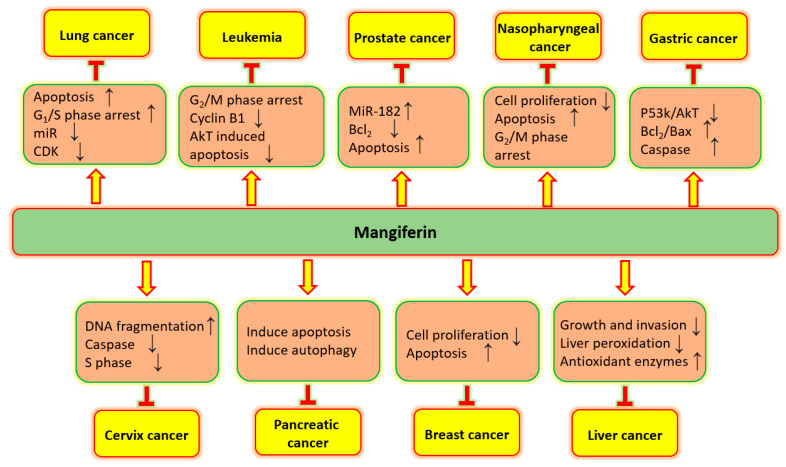
Role of mangiferin in inhibition of different cancers through regulation of cell signaling pathways.

Mangiferin established its role in anti-cancer potential in numerous types of cancer via controlling many cell-signaling molecules (Figure 4). Potential role of mangiferin in several types of cancers are described below:

### 4.1. Lung Cancer

As per the statistics of GLOBOCAN, it accounted for 2.1 million new cases and 1.8 million deaths and is the chief cause of cancer-associated death worldwide [79,80]. Non-small-cell lung cancer, the most common lung cancer histology, is a heterogeneous malignancy including molecular subtypes for which targeted agents are accessible in clinical practice [81]. Different procedures like surgical invasion, radiotherapy, and chemotherapy are presently the main clinical treatments for this malignancy [82,83].

A549, H1299, and H2030 lung cancer cells were found to be restricted in their ability to proliferate by mangiferin, dependent on dosage and time of exposure. Mangiferin also has the ability to trigger apoptosis, and its exposure caused more G1- and S-phase cell arrests than its absence did in untreated cells. Microarray and micro-RNA sequencing studies suggested that lung cancer tissues have higher levels of miR-92a and miR-27b than non-lung adenocarcinoma tissues. Additional research indicated a possible link between mangiferin and lower levels of miR-92a and miR-27b. To examine the potential effect of mangiferin on the miR-92a and miR-27b levels, cells (H1299 and H2030 cells) were treated with mangiferin (25 µM), and then the levels of miR-92a and miR-27b were identified. The outcomes advised that miR-92a and miR-27b levels were evidently diminished in H2030 and H1299 cells after 24 h of mangiferin treatment [84] (Table 2).

Wei Shi et al. reported that in vitro studies established that mangiferin showed apoptosis-induction and growth-inhibitory effects against A549 human lung adenocarcinoma cells. Mangiferin also showed anti-tumor activities in A549 xenograft mice, according to in vivo research. Inhibition of the PKC-NF-kB pathway and activation of G2/M-phase arrest through downregulation of the cyclin-dependent kinase 1-cyclin B1 signaling. Mangiferin treatment promoted apoptotic cell death. The possibility for a combined therapy was also shown by the ability of mangiferin to enhance the antiproliferative capacity of cisplatin in A549 cells. Surprisingly, assessment of body weight confirmed that after treatment with various doses of mangiferin for 14 days the weight of the mice decreased (*p* < 0.05) [28] (Table 2). Transferrin-modified mangiferin-loaded solid lipid nanoparticles were evaluated. It was reported that Tf-MGF-SLNs were extremely effective in suppressing tumor growth in a xenograft tumor model. This formulation would be a hopeful formulation for the treatment of lung cancer [85].

Weight of lungs, and levels of xenobiotic and hepatic marker enzymes were noticeably enhanced, and body weight was reduced in the carcinogen-given animals, and mangiferin treatment played a role in such changes. Further, histopathological-based findings reported that control-group animals showed normal architecture cells and the lung-cancer–bearing-animal group showed alveolar damage, loss of architecture and irregular nuclei in the cells of the alveolar wall. Moreover, cancer-bearing animals pretreated with mangiferin presented decreased alveolar damage with almost normal architecture [86].

**Table 2:** Effects and mechanism of mangiferin in different cancers.

Cancer	Study Types	Outcome of Study	Refs.
Lung	A549, H1299 and H2030	Mangiferin controls the proliferation of adenocarcinoma cells and initiates apoptosis.	[84]
	A549	Mangiferin showed apoptosis-induction and growth-inhibitory effects. It was capable of increasing the anti-proliferative potential of cisplatin.	[28]
Breast	MDA-MB-231	Following the administration with mangiferin, this cancer cell decreased Rac1/Cdc42, WAVE2, phospho-Rac1/Cdc42, Arp2, and Arp3.	[87]
	MCF7 and MCF10A	Mangiferin promotes apoptosis and stops cell proliferation.	[88]
	MCF-7	Mangiferin addition to doxorubicin has a tendency to enhance the sensitivity of the cells to doxorubicin. Adding mangiferin to doxorubicin somewhat reduced the level of expression of P-gp mRNA.	[89]
Cervix	HT29 and HeLa	Mangiferin decreased oxaliplatin IC50 values in cancer cells. DNA fragmentation, enhanced caspase 3 activation, retarted S-phase of cell cycle.	[41]
Prostate	PC3	Mangiferin treatment reduced cancer cells proliferation. Its administration was found to induce apoptosis and enhance the activity of caspase-3.	[29]
Colorectal	Allograft mouse model of murine CT26	Mangiferin treatments brings dose-dependent tumor regression and reduces metastasis. This compound inhibits tumour growth, angiogenesis, and metastasis.	[90]
Liver	Diethynitrosamine-Induced Hepatocellular Carcinoma	Mangiferin shows anticarcinogenic properties against this carcinoma.	[91]
	HCC implantation murine model MHCC97L and HLF	Delay in G1/S transition was dependent on the amounts of mangiferin administered to HCC cells.	[92]
Gastric	SGC-7901	Elevation in Bad, Bax, and cleaved caspase-3,-9 and decrease in Mcl-1, Bcl-xL, and Bcl-2 activities was noticed by mangiferin. In addition, mangiferin lowered p-PI3K, p-mTOR, and p-Akt quantities.	[31]
Brain	U-87 MG and U-118 MG	Mangiferin enhanced the radiosensitivity of cancer cells towards radiation. Cancer cells treated with mangiferin revealed a greater amount of DNA damage, particularly corresponding to the elevated degree of radio sensitization.	[93]
	U373MG, U87MG and CRT-MG	Inhibition of MMP-9 encouraged by mangiferin is associated with the suppression of glioma cell invasion.	[71]
Ovarian	ES-2 and A2780	The proliferation of cancer cells was suppressed by mangiferin. This compound decreases both the cancer cell invasion and migration.	[94]
Bone	Saos-2 and U2OS	Mangiferin decrease the cancer cell viability and proliferative potential.	
Oral	7, 12-dimethylbenz [a] anthracene induced oral cancer	Orally administered mangiferin effectively prevented body weight gain and tumour progression.	[95]
Thyroid	TPC-1	Viability of TPC-1 cells was decreased by mangiferin in a dose-dependent manner and mangiferin brings apoptosis.	[96]
Head and neck	CNE2	Mangiferin inhibits cancer cell proliferation through induction of early apoptosis and G2/M arrest. Furthermore, mRNA and protein levels of Bax were up-regulated and Bcl-2 was to be down-regulated.	[62]
Blood	HL-60	Mangiferin stops cancer cell growth, and cells in the G2/M stage increased in number, and the G2/M phase was arrested.	[27]
	HL-60	Mangiferin led to a decrease in the NF-κB p65 and suppressed the expressions of Bcl-xL as well as XIAP.	[97]
	HL-60	Mangiferin increases the accumulation of the Nrf2 protein in HL-60, primarily in the nucleus.	[35]
Multiple myeloma	IM9 cells, RPMI8226 and RPMI1788	Mangiferin caused a decrease in the mitochondrial membrane potential and increased the number of apoptotic cells.	[36]
	IM9 cells and RPMI8226	Mangiferin in combination with an anti-cancer agent decreased the viability of multiple myeloma stem cell lines.	[98]
Pancreas	Mia-PACa2	Mangiferin increased the expression of LC3 II; in addition, Beclin-1, Bcl-2 decreased, and Bax expression increased dose dependently.	[99]

### 4.2. Breast Cancer

One of the most prevalent malignancies for women is breast cancer, and it is an important public health concern globally [100]. The survival rate of breast cancer patients is still low. The current mode of treatment causes adverse effects on health. Natural compound-based treatment plays an important role in this cancer’s prevention. After treatment of MDA-MB-231 and MCF-7 tumor cells with mangiferin, it was noticed that induction took place as dose-responsive inhibition in growth as compared to the control groups. After 24 h of treatment, mangiferin brought a dose-responsive decrease in the viability of MCF-7 and MDA-MB-231 breast cancer cells as compared to control cells. A dose-responsive drop in Rac1/Cdc42, WAVE2, phospho-Rac1/Cdc42, Arp2, and Arp3 was seen in MDA-MB-231 mammary tumor cells after 4 days of mangiferin therapy, in contrast to cells in respective control groups, according to a Western blot analysis. Furthermore, treatment with mangiferin (10 μM) caused almost complete removal of positive WAVE2 immunofluorescent staining. In contrast to cells in the correspondingly treated control groups, mangiferin treatment resulted in a noticeably lower amount of positive WAVE2 staining in MDA-MB-231 breast tumor cells. After treatment of MDA-MB-231 and MCF-7 tumor cells with mangiferin, it was noticed that induction resulted in a dose-responsive inhibition in growth as compared to the control groups [87]. Mangiferin was capable of selectively stopping the growth of breast cancer cells through prompting apoptosis and via arresting cell proliferation [88] (Table 2). According to another study’s findings, the addition of mangiferin to doxorubicin has the potential to increase the cells’ sensitivity to the drug after 96 h of drug delivery to MCF-7 cells that have already had doxorubicin treatment. The MCF-7 cell count did not significantly decrease with mangiferin (10 and 25 µM), but it did with mangiferin (50 µM) [89].

### 4.3. Cervical Cancer

Apoptosis was promoted in human cervical cancer HeLa cells by the ethanolic extract of mango peel, as reported by the rise in the sub-G1-phase cells [101]. How oxaliplatin and mangiferin anti-cancer potential worked together was investigated. Oxaliplatin IC50 values in HT29 and HeLa cells were lowered by the addition of mangiferin (10 µg/mL). This was followed by increased DNA fragmentation, increased caspase 3 activation, and a delay in the cell cycle’s S-phase. In addition, it was shown that mangiferin inhibited NF-kB activation in HT29 cells that were less resistant to oxaliplatin. The current study indicates that mangiferin supports apoptotic cell death when combined with oxaliplatin, increasing oxaliplatin’s in vitro effectiveness [41] (Table 2).

### 4.4. Prostate Cancer

Middle-aged men between 45 and 60 years of age can suffer from prostate cancer, and this is the uppermost cause of male cancer-associated mortalities [102]. Mangiferin therapy on human prostate cancer cells was investigated for its antitumor potential. Reduction in the proliferation of prostate cancer cells was noticed after mangiferin treatment. Mangiferin also noted that prostate cancer cells were promoted to undergo apoptosis and had their caspase-3 activity boosted. Mangiferin administration significantly enhanced miR-182 expression and decreased Bcl-2 expression levels in prostate cancer PC3 cells. In conclusion, it was found that mangiferin increased the apoptosis in PC3 cancer cells. The observed effect corresponded to miR-182 upregulation and Bcl-2 downregulation. Caspase-3 activity of prostate cells treated with 20 and 40 µM mangiferin was significantly enhanced as compared with that of the control group. To further understand the association between miR-182 levels and the anti-proliferative action of mangiferin, miR-182 inhibitor was transfected into cancer cells. The transfection with miR-182 inhibitor drastically reduced miR-182 expression levels in these cells. However, the miR-182 inhibitor was also able to decrease the anti-proliferative and apoptotic effects of mangiferin (50 µM). Thus, miR-182 inhibitor could have potential for rescuing Bcl-2 expression in PC3 cells [29] (Table 2).

### 4.5. Colon Cancer

In a syngeneic mouse model of murine CT26 colon cancer, a recent study finding showed that treatment of colorectal cancer with the drug mangiferin results in dose-dependent tumor regression and lowers lung metastasis, improving mouse survival in general. Surprisingly, transcriptome pathway enrichment evaluation demonstrates that mangiferin inhibits tumor growth, metastasis, and angiogenesis. By focusing on mitochondrial energy metabolism in the tumor microenvironment, mangiferin antiangiogenic, anti-cancer, and antimetastatic activities minimize the therapeutic result of colorectal cancer treatment [90] (Table 2). Another study conducted two experiments: an intermittent analysis to look at how mangiferin affected the development of preneoplastic lesions caused by azoxymethane, aberrant crypt foci (ACF), and a follow-up long-term investigation to explore how mangiferin affected carcinogenesis. In the short-term assay, it showed that rats receiving azoxymethane were provided with 0.1% mangiferin in their diets, which significantly reduced the development of aberrant crypt foci in contrast to rats given azoxymethane alone. The group that received 0.1% mangiferin throughout the experimental protocol’s initial stages displayed a noticeably decreased incidence and multiplicity of intestinal neoplasms brought on by azoxymethane in the long-term evaluation. Mangiferin treatment significantly (*p* < 0.01 or *p* < 0.05) inhibited ACF induced by AOM by 40%, aberrant crypts in ACF in the colon by 43%, and the total foci number with four or more crypts by 52%. Furthermore, the colonic mucosa cell proliferation was decreased in animals treated with mangiferin [103].

### 4.6. Liver Cancer

The anticarcinogenic potential of mangiferin against DEN-caused hepatocellular carcinoma was investigated. The anticarcinogenic property of mangiferin was established by assessing the apoptotic protein expression and histological analysis of liver tissue from DEN- and DEN-plus-mangiferin-administered rats. Results demonstrated that mangiferin holds anticarcinogenic properties against DEN-caused hepatocellular carcinomas. Further, a histopathological-based study’s findings revealed that livers of control rats and mangiferin-treated rats demonstrated normal architecture, while increased tumor nodules were noticed in the DEN-treated group of rats [91] (Table 2).

Orthotopic tumor growth was inhibited by oral administration of mangiferin. Mangiferin has been shown in cellular studies to inhibit the growth and invasion of hepatocellular carcinoma in a way that is dose-dependent. Mangiferin primary target was the Wnt pathway, and LEF1 was the Wnt pathway gene that had been lowered the greatest. In hepatocellular carcinoma cells administered mangiferin, overexpression of LEF1 reduced the suppression of Wnt signaling and also lowered proliferative activity. On the other hand, WT1 protein was associated with the mangiferin-mediated reduction of LEF1 that was not dependent on β-catenin [92]. Another study finding reported that mangiferin treatment prevents lipid peroxidation, liver damage, and protects the antioxidant defense system in DEN-caused liver carcinogenesis in rats [104].

### 4.7. Gastric Cancer

Gastric cancer is a multifactorial disease, where numerous factors can affect its development, including both environmental as well as genetic factors [105]. Min Du et al. demonstrated that mangiferin treatment played an important role in the elevation of Bad, Bax, cleaved caspase-9, and caspase-3 expression and in the decrease in Bcl-2, Bcl-xL, and Mcl-1 expression in gastric cancer cells. Furthermore, mangiferin meaningfully reduced the degree of p-PI3K, p-Akt, and p-mTOR, but showed no role in their non-phosphorylated forms in SGC-7901 cells that were exposed to epidermal growth factor. Remarkably, the proapoptotic role of mangiferin in the utilized cancer cells was partly stopped by the Akt activator SC79, while LY294002 meaningfully enhanced mangiferin-caused apoptosis as well as growth inhibition. Results show that mangiferin successfully prevents gastric cancer cells from growing and starts the process of apoptosis by obstructing the PI3K/Akt pathways [31] (Table 2). Co-culturing of Helicobacter pylori-infected AGS cells with mangiferin significantly enhanced the inhibitory zone as well as noticeably reduced the content of MBC and MIC, together with a dose-dependent reduction in adhesion and invasive activity (*p* < 0.05). In the meantime, inflammatory markers such as TNF-α interleukins-1β, IL-8, NF-κΒ, and subunit p65 were further noticeably suppressed with mangiferin administration. In addition, AGS cells exposed to mangiferin showed a notable reduction in the protein production of inflammatory enzymes such as iNOS and COX-2 (*p* < 0.05) [26].

### 4.8. Brain Cancer

Among the various cancer types, brain cancers have one of the lowermost incidences; however, they are amongst the most invasive as well as with the highest mortality rates [26]. According to a recent study, mangiferin might make glioblastoma cells more sensitive to radiation. As a result, MTT assays were carried out using U-118 MG and 87 MG cells in order to verify this assumption and explore the inhibitory role of mangiferin in glioblastoma cells after IR. Mangiferin was suggested to be a potential radiosensitive agent for the therapy of glioblastoma after IR, as evidenced by the significantly reduced growth rates of U-87 MG cells that received 25 µg/mL mangiferin after 5-Gy IR compared to those of mock-treated cells. Furthermore, after receiving a combination of mangiferin and IR, the growth rates of U-118 MG cells were significantly inhibited (*p* < 0.05). These findings collectively suggested that mangiferin might increase the radiosensitivity of glioblastoma cells to radiation and hence reduce the viability of cells after radiation. Mangiferin exposure also increased the percentages of DNA damage in the glioblastoma cells (U-87 MG cells and U-118 MG cells), which corresponds to a substantial amount of radio-sensitization (*p* < 0.05). Due to the inhibition of DNA-damage repair, our findings indicated that mangiferin most likely mediates radiosensitization. Additionally, through in vivo tumor-bearing mouse tests, mice given mangiferin after radiation showed reduced tumor weight, a smaller tumor volume, and an extended life span [93] (Table 2).

It was found that mangiferin precisely prevented MMP-9 gene expression in phorbol myristate acetate (PMA)-stimulated human astroglioma U373MG and U87MG, CRT-MG cells. MMP-9 expression at the promoter, mRNA, and protein levels were suppressed by mangiferin and prevented MMP-9 enzymatic activity. Additionally, mangiferin appears to be linked to MMP-9 inhibition via initiating the suppression of glioma cells’ in vitro invasiveness. Mangiferin targeted the inhibition of MMP-9 and may, therefore, provide an effective pharmacological strategy for treating gliomas [71].

### 4.9. Ovarian Cancer

One of the most lethal gynecological malignancies is ovarian cancer with a 5-year survival rate of only 17% during the advanced stages [106]. Mangiferin inhibited epithelial ovarian cancer cell proliferation. Additionally, treatment with progressive dosages of mangiferin severely reduced the motility of ES-2 and A2780 cells in affected areas compared to the control. Mangiferin may also, in a dose-dependent manner, reduce cancer cell invasion as well as migration from upper to lower chambers. Further in vivo results revealed that there was a clear difference between the mangiferin and control groups in the weight and volume of the isolated tumors. The high dose of mangiferin (60 mg/kg) led to a more efficient prevention of tumor growth when compared to a lesser dose of 20 mg/kg, suggesting that 60 mg/kg mangiferin was the optimal dose in vivo [94] (Table 2).

### 4.10. Bone Cancer

Osteosarcoma, a very hostile bone tumor presents a dismal prognosis for poor responders to therapy and for metastasis-presenting subjects [107]. An important study’s result’s reported that mangiferin decreases the Saos-2 and U2OS cell viability. Mangiferin decreased the proliferative potential of Saos-2 cells and reduced the proliferative activity of U2OS cells. After being exposed to different mangiferin doses, Saos-2 cells became non-viable. In contrast, U2OS cells became non-viable after being exposed to different doses of mangiferin, respectively (*p* < 0.05) [108].

### 4.11. Oral Cancer

Min Lui et al. reported that a noteworthy decrease in body weight was noticed in DMBA-induced oral cancer, whereas oral treatment with mangiferin (50 mg/kg bw) led to a substantial improvement in body weight in dimethylbenz[a]anthracene (DMBA)-challenged hamsters. Oral pre-administration of mangiferin alone in hamsters led to hamsters showing the same body weight when compared with the untreated normal group. Moreover, mangiferin with DMBA-treated hamsters significantly reduced the incidence, volume as well as burden of tumors. The body weight, progression of tumors, histological changes, and biochemical changes in DMBA-painted hamsters that were given oral treatment with mangiferin at an effective dosage of 50 mg/kg bw were all purposefully avoided. Furthermore, pretreatment with mangiferin for DMBA-challenged hamsters showed important near-normal levels of non-enzymatic antioxidants to both buccal and mucosa plasma as compared to the control group; moreover, hamsters treated with mangiferin alone and the control controlled no modifications in nonenzymatic antioxidant function [95] (Table 2).

### 4.12. Thyroid Caner

The sustainability of TPC-1 cells was decreased by mangiferin in a dose-dependent way. In addition, mangiferin induced the fragmentation of DNA at both 2 and 4 µM concentrations. Mangiferin brings apoptosis via the induction of cas-3 as well as decreased Bcl-2 expression. Mangiferin was found to activate apoptotic pathways and reduce the viability of TPC-1 cells via inhibiting PCNA [96] (Table 2).

### 4.13. Nasopharyngeal Cancer

A study based on nasopharyngeal cancer was performed to examine the anti-proliferative mechanism. Different doses of mangiferin (12.5, 25, 50, 100, 150, and 200 µM) were applied to the CNE2 cells. Flow cytometry assays’ base result exhibited that mangiferin inhibits CNE2 cell proliferation through induction of early apoptosis and G2/M arrest. In addition, when CNE2 cells were treated with mangiferin, Bax mRNA and protein levels increased, but Bcl-2 levels decreased. This study identified the anti-proliferative effects of mangiferin through the production of cell death controlled by the expression of Bcl-2 and Bax [62].

### 4.14. Leukemia

The role of mangiferin on cycle-regulating genes in leukemia HL-60 cells was investigated as this compound inhibited the proliferation of these cells. After 24 h of therapy, more cells entered the G2/M phase, and an increase in the expression of the genes Wee1, CDC25C, and Chk1 suggested that the G2/M phase had been arrested. Furthermore, mangiferin lowered cdc25c, Ak t, and cyclin B1 protein levels [27] (Table 2).

Wee1 mRNA expression was enhanced by mangiferin treatment, while it inhibited Chk1 and cdc25c mRNA expression at high concentrations. DNA fragmentation and an increase in caspase-3 activity were seen at the same time. Mangiferin also reduced the nuclear entrance of NF-kB p65. Mangiferin also decreased the expression of Bcl-xL and XIAP while maintaining the levels of Bax, Bcl-2, and Bim. The concentration of 50 µM DMF was administered to the HL-60 cells to inhibit the nuclear entry of NF-κB p65. The growth of HL-60 cells meaningfully reduced at 3 days after administration. It was noticed that DMF inhibited the nuclear entry of NF-κB p65 and the expression of XAIP and Bcl-xL. These outcomes designate that mangiferin prompts apoptosis via suppressing the nuclear entry of NF-κB p65 as well as the expression of XIAP and BclxL [97].

Mangiferin (50 µmol/L) enhanced the accumulation of Nrf2 protein in HL-60 cells, primarily in the nucleus. Mangiferin also improved expression level of NQO1 and reduced intracellular ROS via increasing the binding of Nrf2 to an ARE. Mangiferin alone dose-dependently inhibited proliferation in HL-60 cells. In these cancer cells, mangiferin (50 µmol/L) did not reduce the cell toxicity generated by etoposide, and the combination of mangiferin with etoposide actually increased the rate of cell inhibition [35] (Table 2).

In leukemia mice, mangiferin significantly enhanced the body weight and decreased the spleen and liver weights [109].

According to study findings, mangiferin (25–200 µmol/L) reduced the proliferation of the K562 cell line and caused apoptosis. When K562 cells were treated with various amounts of mangiferin, Bcr/abl gene expression was reduced. Mangiferin considerably slows down the growth of leukemia cells, and induces apoptosis in the cancer cell line.

Mangiferin might prevent the telomerase activity of leukemia K562 cells in a time and concentration-dependent way. In the meantime, it might induce apoptosis clearly and enhance the levels of Fas in K562 cells [110]. Mangiferin inhibits telomerase activity of these cancer cells, and the mechanism of outcome is possibly related to acquiring apoptosis and the Fas protein expression [111]. A recent study looked into how mangiferin affected leukemia in BLAB/c mice that had been produced from WEHI-3 cells. In mice suffering from leukemia, mangiferin boosted their body weight and reduced liver and spleen mass. Mangiferin also enhanced the population of CD3 T-cell and CD19 B cell, whereas it reduced the population of Mac-3 macrophages as well as CD11b monocyte. Mangiferin further improved the survival rate of leukemia mice at 40 and 120 mg/kg doses [109].

### 4.15. Multiple Myeloma

Multiple myeloma (MM) accounts for 1.8% of all known cancers with an estimated 32,270 new cases and 12,830 deaths in the USA in 2020 [112]. The molecular pathway responsible for the apoptosis induced by mangiferin was studied in several myeloma cell lines. The findings revealed that mangiferin reduced the viability of multiple myeloma cell lines. The reduction in mitochondrial-membrane potential and increased number of apoptotic cells meant that caspase-3 activation was noticed. Additionally, NF-κB (nuclear factor kappa B)’s nuclear translocation was decreased, and phosphorylated inhibitor kappa B (IκB) was inhibited by mangiferin, and it increased the expression of IκB protein. However, mangiferin treatment did not change the JNK1/2 (c-Jun N-terminal protein kinase 1/2), phosphorylation levels of extracellular signal-regulated kinase 1/2 (ERK1/2), and mammalian target rapamycin (mTOR). Mangiferin also decreased the expression of the proteins Bcl-xL, survivin, and the X-linked inhibitor of the apoptosis protein (XIAP). These findings suggest that mangiferin promotes apoptosis by inhibiting NIK activity in MM cell lines, which in turn inhibits NF-B nuclear translocation [36]. The impact of combining mangiferin with traditional anti-cancer medications in a multiple myeloma cell line was investigated. Multiple myeloma cell lines were reported to be less viable due to a combined administration of mangiferin and an anti-cancer drug as compared to individual treatment. Mangiferin and an anti-cancer treatment decreased cell viability, which was attributed to increased Noxa and p53 expression and decreased XIAP, Bcl-xL, and survival protein expression via inhibiting the NF-kB pathway [98] (Table 2).

### 4.16. Pancreatic Cancer

Pancreatic cancer is a leading cause of death, with a high mortality rate and which occurs due to several reasons [99]. But such a treatment module causes adverse effects. A study based on pancreatic cancer reported that Mia-PACa2 cells were transfected with GFP-LC3 vectors and exposed to different doses of mangiferin. This exhibited an increase in the expression of the LC3, proposing that mangiferin induced autophagy in the Mia-PaCa2 cells. Furthermore, mangiferin also increased the expression of LC3 II and Beclin-1, confirming that the mangiferin caused autophagy. Moreover, the role of mangiferin was examined on the Bax and Bcl-2 expression, which are measured as important biomarker proteins of apoptosis. The outcomes of the immunoblot analysis exhibited that Bcl-2 decreased and the Bax expression increased dose dependently. Furthermore, mangiferin caused important enhancements in the ROS levels of the MiaPaCa2 cells and these properties were found to be concentration dependent, and mangiferin inhibited the migration [57] (Table 2).

## 5. Synergistic Effects of Mangiferin with Other Therapeutic Agents in Cancer Cells

**Figure 5:**
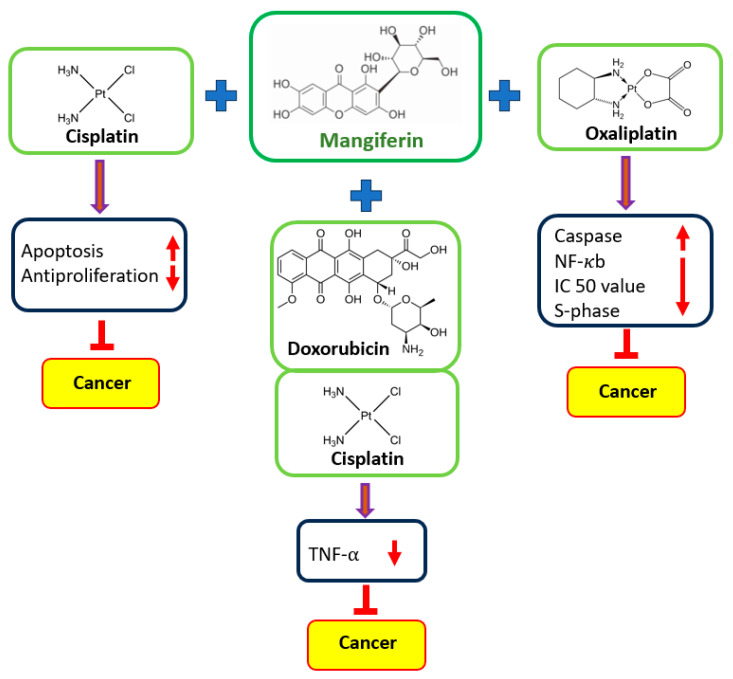
Synergistic effects of mangiferin in combination with cancer drugs.

Combination therapy kills the cancer cells and regulates various gene activities. The synergistic effects of mangiferin in combination with cancer drugs have been shown (Figure 5).

Synergistic effects of mangiferin in combination with other therapeutic drugs in different cancer is described as follows:

### 5.1. Acute Myeloid Leukemia

Acute myeloid leukemia (AML) occurs in hematopoietic progenitor cells of immature myeloid blast cells [113]. Mangiferin (50 μmol/L) enhances Nrf2 protein expression in leukemia HL-60 cells. This compound exclusively inhibited the growth of cancer cells using dose-dependent mechanisms. Mangiferin did not inhibit the cytotoxicity induced by etoposide in HL-60, and when in combination with etoposide, it increased the rate of cell inhibition. Mangiferin does not reduce etoposide-induced apoptosis in these leukemia cells. Moreover, mangiferin meaningfully relieved oxidative stress, whereas it attenuated etoposide-induced cytotoxicity [35] (Table 3).

### 5.2. Colorectal Cancer

The study investigates the combined anti-cancer action of oxaliplatin and mangiferin on cell cycle analysis of HT29 cells. This compound exhibited a delay in the S-phase in combination with oxaliplatin, while no effect was observed on its own apart from enhancement in the subG1 population from 2.6 to 6.2%. The percent of apoptosis was also increased to 8.4% in the mangiferin/oxaliplatin combination. The NF-κB assay showed that oxaliplatin-resistant HT29 cells exhibit an increased amount of activated NF-κB compared to normal HT29 cells. Consequently, mangiferin has been revealed to decrease the amount of activated NF-κB associated with resistant tumor cells and may prove useful in the prevention of resistance to platinum anti-cancer drugs in tumor cells [41].

### 5.3. Cervical Cancer

The combination therapy of mangiferin and oxaliplatin on cervical HeLa cells reported that mangiferin increases the effect of oxaliplatin on these cells. An increase in the percentage of activated caspase-3, reveals that mangiferin certainly enhanced caspase-3 activation in combination with oxaliplatin [41] (Table 3).

### 5.4. Breast Cancer

The study aimed to examine the role of mangiferin to re-sensitize MCF-7 breast cancer cells earlier treated with short-term doxorubicin via the modulation of efflux transporters, MRP1, P-glycoprotein, and BCRP. At low doses of mangiferin (10 and 25 μM), doxorubicin did not reduce cell viability. However, at higher doses, this combination reduces the viability of cells. It shows that mangiferin can be employed as a chemosensitizer for doxorubicin at higher dosages [102] (Table 3).

### 5.5. Colon Cancer

The antiproliferative effects of mangiferin alone and in conjunction with modest dosages of chemotherapeutic drugs were studied. In the experimental setting of this investigation, combining mangiferin with low, non-cytotoxic quantities of cisplatin and 5-fluorouracil promotes cell death and was found to increase the cytotoxicity of these chemotherapeutic drugs [14] (Table 3).

### 5.6. Lung Cancer

In vitro studies established that mangiferin showed apoptosis-inducing and growth-inhibitory effects against lung cancer A549 cells. Furthermore, mangiferin showed anti-tumor potential in A549 xenograft mice in vivo. The potential for combination therapy was further demonstrated by the capacity of mangiferin to uplift the antiproliferative effects of cisplatin on lung cancer A549 cells [28] (Table 3).

## 6. Approaches to Improve the Mangiferin Delivery

The mangiferin has proven to have a therapeutic role in disease curing including cancer through modulating cell-signaling molecules. Even though there are important potential health benefits of mangiferin, this compound shows considerable limitations for its clinical use due to its poor oral bioavailability (1.2%) [114], and its water solubility is 0.111 mg/mL [115]. The delivery carriers based on a nano-formulation or structural modification method have been used to enhance the efficacies of this compound. Moreover, to recognize more effective therapeutic compounds, mangiferin derivatives with better solubility, bioavailability, and effectiveness were attained using chemical or biotransformation approaches [116].

Some formulation based on mangiferin is discussed here, as kind of poly (lactic-co-glycolic acid) (PLGA) NPs loaded with mangiferin were synthesized to evaluate their anti-topoisomerase activity. Anti-topoisomerase assay exhibited that the optimal formulation showed anti-proliferative potential. The results showed that even at 2500 µg/mL concentration, MG4 had non-cytotoxic effects on HEPG2 and BEAS 2B. In conclusion, this investigation demonstrated an encapsulation method that was resistant to in vitro gastric digestion while also not impairing the biological function as well as metabolism of healthy cells [117]. Further, mangiferin-enriched gold nanoparticulate (MGF-AuNPs) exhibits immunomodulatory properties that can treat prostate cancer. The discovery of higher concentrations of anti-tumor cytokines and a simultaneous reduction of pro-tumor cytokines presented evidence for the immunomodulatory action of MGF-AuNPs in prostate tumors. TNF-α and IL-12 levels were both increased in the MGF-AuNPs-treated groups by roughly 50 and 10 fold, respectively, while IL-10 and IL-6 levels were both decreased by 2 fold. Hence, the therapeutic efficiency of MGF-AuNPs in treating prostate malignancy in vivo in tumor-bearing mice takes into consideration of several immunomodulatory interventions activated by this green nanotechnology-based nanomedicine agent [118]. Comprehensive in vivo therapeutic efficiency studies, through the intratumoral delivery of MGF-^198^AuNPs, display the retention of over 80% of the injected dose in prostate tumors up to 24 h. Three weeks post treatment, tumor volumes of the treated group of animals displayed a more than five-fold decrease as compared to the control saline group [119]. Nanoparticles with N,O-Carboxymethyl Chitosan-Mangiferin were created, and their antioxidant and cytotoxic properties was evaluated. MTT assay was used to assess the cytotoxic capability of mangiferin-N,O-CMC nanoparticles on Osteosarcoma MG-63 and 3T3 cells. In the MTT assay, mangiferin-N, O-CMC nanoparticles meaningfully reduced the growth of osteosarcoma MG-63 cells. The synthesized mangiferin-N,O-CMC nanoparticles were shown to be very efficient nanocarriers for transferring mangiferin to cancer cells, as demonstrated by the aforementioned information [120]. In addition, mangiferin-conjugated carbon nanotubes were created using polyethylene glycol. The IC50 value was found to be decreased by 1.28-fold, suggesting strong anti-cancer potential, according to these cytotoxicity-based observations. Flow cytometry-based findings demonstrated effective apoptosis induction with the least necrosis via the nano-conjugate vis à vis the naïve drug. From these discovered outcomes, it can be reported that these functionalized nanocarriers are able to offer the powerful, as well as safer, delivery of phytochemicals to the brain cancerous cells [121].

## 7. Clinical Studies on Mangiferin

Many studies have emphasized the potential health benefits of mangiferin, including its mechanisms against numerous cancers. Even though there are significant potential health benefits of mangiferin, this compound shows substantial restrictions for its clinical use due to its low oral bioavailability (1.2%) [114], and its water solubility is 0.111 mg/mL [115]. Even though *mangiferin* has not been assessed for clinical studies for cancer treatment, some clinical trials are ongoing or are completed based on mangiferin in other pathogenesis, and it is anticipated to become an auspicious therapeutic remedy in cancer treatment. Some clinical studies have assessed the role of mangiferin on serum lipid profiles in overweight patients with hyperlipidemia. These patients were involved in this double-blind randomized controlled trial. Mangiferin supplementation significantly increased the serum levels of HDL-cholesterol, β-hydroxybutyrate, L-carnitine, and acetoacetate. This compound could also recover serum lipid profiles by lowering serum TGs and FFAs in patients with hyperlipidemia, due to the promotion of free fatty acids oxidation [122]. A mango juice by-product reduced the incidence of gastrointestinal and upper-tract respiratory problems and such benefits were associated with increased serum levels of PAI-I, MIP-1a, and MIP-1b [123].

## 8. Conclusions, Challenges and Future Prospective

Cancer accounts for a substantial proportion of mortality worldwide and is a significant burden on the healthcare system. Treatment modules like surgery, chemotherapy, and radiotherapy are used to treat cancer, but still cancer is a major problem related to deaths worldwide. However, safe and effective treatment modules are needed to overcome the adverse effects. Several studies demonstrated that natural products could influence cell-signaling molecules in managing cancer.

Mangiferin is a xanthone, and its ability to counteract inflammation and free radicals has made it useful in the management of health. The potential mechanisms of mangiferin as an anti-cancer potential include inhibiting cancer cell proliferation, oxidative stress, inflammation, and angiogenesis, inducing apoptosis, and cell-cycle arrest. Furthermore, synergistic effects with anti-cancer drugs including etoposide, oxaliplatin, doxorubicin, cisplatin, and 5-fluorouracil has been reported through the enhancement of anti-cancer drug efficacies via modulating various cell-signaling processes.

Even though there are very significant potential health benefits of mangiferin, this compound shows substantial restrictions for its clinical use in cancer management due to poor water solubility, low absorption and rapid elimination [124]. Mangiferin led to an exhaustive hepatic first-pass metabolism, reducing the fraction of the dose reaching the systemic circulation, which is a reason causing the low oral bioavailability, leading to limitation of the efficacy [125]. An HPLC/MS method for the measurement of mangiferin in rat plasma was performed. The method was confirmed over the concentration range 0.02–5.0 microg/mL for oral administration, as well as 0.4–100 micro g/mL for intravenous administration [114]. Moreover, oral absorption of mangiferin from rats is pretty low with bioavailability of only 1.2% [126], and the maximum plasma levels are fairly low and unpredictable; in other words, 715.04 ± 600.14 ng/mL was obtained after 0.72 h of its oral intake [126].

There are some areas such as bioavailability and safety level where more research is required to be explored to better our knowledge of mangiferin and its possible implications for human health management. More studies need to be performed to understand the mechanism of action, safety level, and its optimum therapeutic dosage.

Further, extensive research of mangiferin in vivo and in vitro has been performed to evaluate its role in cancer management, but a lack of robust clinical data/clinical trials limit its usefulness and acceptance in humans. More clinical trials should be performed to reconnoiter the anti-cancer potential in human. Considering the challenges of poor bioavailability, nano-formulations-based delivery systems should be performed to increase the solubility, bioavailability, and efficacy of this compound. Overall, this study suggests that mangiferin can be used as a therapeutic remedy in inhibiting cancer or improving clinical symptoms due to its anti-inflammation and anti-antioxidant potential.

## Figures and Tables

**Table 1:** Role of mangiferin in cancer prevention through different mechanisms of action.

Action on	Cancer	Cell Line	Dose	Outcome	Refs.
Inflammation	Gastric	AGS	10, 20, 50 & 100 μg/mL	iNOS protein expression as well as COX-2 downregulated with increased concentration of mangiferin	[26]
Cell cycle	Blood	HL-60	20–250 µM	Mangiferin alters cell cycle arrest at the G2/M phase.	[27]
	Lung	A549	25 µg/mL	Mangiferin administration, more cells were arrested in the sub-G1 phase	[28]
Apoptosis	Prostate	PC3	20 & 40 µM	Apoptosis of prostate cancer cells was increased by mangiferin treatment	[29]
	Ovarian	Ovcar3	25 µg/mL	Cells treated with mangiferin showed membrane blebbing, shrinkage of cytoplasm and nucleus	[30]
PI3K/Akt	Gastric	SGC-7901	5 & 10 µmol/L	Expression of p-mTOR, p-PI3K, and p-Akt, were lowered by mangiferin treatment.	[31]
Metalloproteinase	Prostate	LNCaP	400 µM	Cell stimulation with TNF-α enhanced MMP-9 expression; while mangiferin suppressed this effect	[32]
	Breast	MDA-MB-231	12, 25 & 50 µM	Mangiferin was negatively regulate MMP-9 and -7	[33]
Epithelial to Mesenchymal Transition	Breast	MDA-MB-231 and BT-549	12, 25 & 50 µM	Anticancer potential induced by mangiferin via modulation of MMP-7 and -9, and EMT	[33]
Nrf2	Blood	HL60	50, 100 or 200 μM	Dose-dependent increase in the Nrf2 protein level after mangiferin treatment	[34]
	Blood	HL60	50, 100 & 200 mol/L	Mangiferin increased the whole-cell buildup of Nrf2 protein.	[35]

**Table 3:** Synergistic effects of mangiferin with anti-cancer drugs.

Cancer	Cell Lines	Anti-Cancer Drugs/Treatment Type	Outcome of the Study	Refs.
Myeloid leukemia	HL60	Etoposide	Mangiferin decreases the cytotoxicity caused by etoposide in cancer cells, and when combined with a low concentration of etoposide, the treatment even increases the rate of cell inhibition.	[35]
Cervix, breast, and colon cancer	HeLa, HT29, and MCF7	Oxaliplatin	Mangiferin decrease the oxaliplatin IC_50_ values. A prolonged S-phase of the cell cycle, increased caspase 3 activation.	[41]
Breast cancer	MCF-7	Doxorubicin	Cell viability was decreased substantially when doxorubicin was administered in conjunction with mangiferin.	[102]
Colon cancer	CT26.WT	Cisplatin and 5-fluorouracil	Combining mangiferin with 5-fluorouracil and cisplatin promotes cell death and the cytotoxicity of drugs.	[14]
Lung cancer	A549	Etoposide and cisplatin	Mangiferin demonstrated the promising potential of the combination therapy by increasing the antiproliferative capacity of cisplatin on cancer cells.	[28]

## Data Availability

Data are contained within the article.
